# FBXO5-mediated RNF183 degradation prevents endoplasmic reticulum stress-induced apoptosis and promotes colon cancer progression

**DOI:** 10.1038/s41419-024-06421-2

**Published:** 2024-01-11

**Authors:** Jing Ji, Aixin Jing, Yuanyuan Ding, Xinhui Ma, Qilan Qian, Ting Geng, Wenhao Cheng, Meiqi Zhang, Qian Sun, Shaojie Ma, Xiujun Wang, Qing Yuan, Menghan Xu, Jingting Qin, Lin Ma, Jiayan Yang, Jingliang He, Qianming Du, Mengbei Xia, Yuting Xu, Ziyun Chen, Lan Zhu, Wei Liu, Shunfang Liu, Bin Liu

**Affiliations:** 1 Jiangsu Key Laboratory of Marine Pharmaceutical Compound Screening, College of Pharmacy, Jiangsu Ocean University, Lianyungang, 222005 China; 2 Cancer Center and Department of Pharmacology and Toxicology, Medical College of Wisconsin, Milwaukee, WI 53226 USA; 3 School of Basic Medicine & Clinical Pharmacy, China Pharmaceutical University, Nanjing, 210009 P.R. China; 4 The First People’s Hospital of Lianyungang, the First Affiliated Hospital of Kangda College of Nanjing Medical University. 7 Zhenhua Road, Haizhou, Lianyungang, 222061 Jiangsu PR China; 5 General Clinical Research Center, Nanjing First Hospital, Nanjing Medical University, Nanjing, 210006 P.R. China; 6 Department of Oncology, Tongji Hospital, Tongji Medical College, Huazhong University of Science and Technology, Wuhan, 430030 China

**Keywords:** Cancer prevention, Cancer prevention

## Abstract

Endoplasmic reticulum (ER) stress induces the unfolded protein response (UPR), and prolonged ER stress leads to cell apoptosis. Despite increasing research in this area, the underlying molecular mechanisms remain unclear. Here, we discover that ER stress upregulates the UPR signaling pathway while downregulating E2F target gene expression and inhibiting the G2/M phase transition. Prolonged ER stress decreases the mRNA levels of E2F2, which specifically regulates the expression of F-Box Protein 5(FBXO5), an F-box protein that functions as an inhibitor of the anaphase-promoting complex/cyclosome (APC/C) ubiquitin ligase complex. Depletion of FBXO5 results in increased ER stress-induced apoptosis and decreased expression of proteins related to PERK/IRE1α/ATF6 signaling. Overexpression of FBXO5 wild-type (not its ΔF-box mutant) alleviates apoptosis and the expression of the C/EBP Homologous Protein (CHOP*)*/ATF. Mechanistically, we find that FBXO5 directly binds to and promotes the ubiquitin-dependent degradation of RNF183, which acts as a ubiquitin E3 ligase in regulating ER stress-induced apoptosis. Reversal of the apoptosis defects caused by FBXO5 deficiency in colorectal cancer cells can be achieved by knocking down RNF183 in FBXO5-deficient cells. Functionally, we observed significant upregulation of FBXO5 in colon cancer tissues, and its silencing suppresses tumor occurrence in vivo. Therefore, our study highlights the critical role of the FBXO5/RNF183 axis in ER stress regulation and identifies a potential therapeutic target for colon cancer treatment.

## Introduction

Endoplasmic reticulum (ER) stress is crucial for maintaining cellular homeostasis and activates the unfolded protein response (UPR) by reducing protein synthesis in response to protein folding reactions, thereby promoting the unfolded protein response [1, 2]. This leads to an increase in chaperone molecules that facilitate protein misfolding, ultimately triggering cell apoptosis [3, 4]. Importantly, ER stress plays a pivotal role in the proliferation and viability of cancer cells, particularly in the context of colorectal cancer where the intestinal epithelium is enriched in ER structures [5]. The UPR in the ER encompasses three pathways: PERK, IRE1α, and ATF6 pathways [6]. Furthermore, ATF4, downstream of the PERK protein, significantly regulates the expression of CHOP [7]. CHOP serves as a stress response to ER stress, and under conditions of severe ER stress, cell apoptosis is initiated with the upregulation of CHOP and subsequent activation of Bcl-2 to facilitate cell apoptosis [8, 9]. Investigating the targets and mechanisms of ER stress and its associated pathways in mediating cell apoptosis holds promise for providing novel insights into the treatment of colon cancer.

FBXO5, a member of the F-box protein family, acts as a specific component of the ubiquitin ligase complex [10]. It interacts with Skp1 protein in other E3 complexes to form the SCF (Skp1-cullin-F-box) complex, which facilitates the ubiquitination of specific target proteins, thereby regulating their degradation and related biological processes [11]. Of particular importance, investigations have unveiled the potent inhibitory role of FBXO5 on the anaphase-promoting complex/cyclosome (APC/C) ubiquitin ligase complex, thereby exerting a critical impact on the cell cycle [12]. This pivotal function of FBXO5 directly influences crucial cellular processes, including cell proliferation, apoptosis, and cell differentiation [13–15]. However, there have been relatively few reports on the involvement of FBXO5 in colorectal cancer thus far.

In our study, we have discovered a novel finding that ER stress can suppress FBXO5 expression by mediating the transcription factor E2F2, ultimately leading to increased cellular apoptosis. Interestingly, in colon cancer, FBXO5 is found to be overexpressed and plays a role in preventing ER stress-induced apoptosis via its ability to ubiquitinate and degrade RNF183, a critical regulator of ER stress-induced apoptosis. These observations suggest a potential role for FBXO5 in the progression of colorectal cancer.

## Material and methods

### Data collection and analysis

Download microarray data from the Gene Expression Omnibus (GEO) database on the NCBI website, specifically GSE41666 (Platform: GPL10558) and GSE62321 (Platform: GPL97). GSE41666 comprises gene expression profiles from HCT116 cells treated with either DMSO or thapsigargin (TG), while GSE62321 includes 20 colon cancer tissue samples and 18 normal colon tissue samples. The expression levels of FBXO5 mRNA in GSE62321 were plotted using Graphpad Prism 9 software. Normalization of the transcriptome data was performed using the “limma” package in R-4.1.3 software, followed by differential analysis (with a *p* < 0.001 and |log2FC | > 1.5 cutoff). The clustering analysis heatmap was generated using the “pheatmap” package, and the volcano plot was created using the “ggplot2” package. Gene Ontology (GO) analysis and Kyoto Encyclopedia of Genes and Genomes (KEGG) analysis were conducted on the downregulated genes from GSE41666 and the 200 co-expressed genes of FBXO5 in TCGA colon tumor tissues. The “EnrichGO” function in the R package “clusterProfiler” was employed for GO analysis, while the “EnrichKEGG” function in the R package “clusterProfiler” was utilized for KEGG analysis. Gene Set Enrichment Analysis (GSEA) was performed using the R packages “fgsea,” “enrichplot,” “ggsea,” and “clusterProfiler,” with the Hallmark gene set downloaded from http://www.gsea-msigdb.org/. Co-expressed proteins of FBXO5 (200 proteins) were obtained from the cBioPortal database (https://www.cbioportal.org/), and an online tool called imageGP (http://www.ImageGP/VennDiagram.html) was used to generate a Venn diagram comparing these proteins with the 489 downregulated proteins in thapsigargin-treated HCT116 cells. RNA-seq data for colon cancer and non-colon cancer tissues were retrieved from the TCGA database, while RNA-seq data for normal colon tissues were obtained from the GTEx database. The correlation between E2F2 and FBXO5 in colon cancer, non-colon cancer and normal colon tissues was analyzed using the GEPIA2 online tool (http://gepia2.cancer-pku.cn), and the Pearson correlation coefficient was calculated. The GEP1A2 website (http://gepia2.cancer-pku.cn) was employed to plot the differential expression profile of FBXO5 in TCGA-COAD. Immunohistochemistry images of FBXO5 in colon cancer cells were retrieved from the HPA database (https://www.proteinatlas.org/). The JASPAR (https://jaspar.genereg.net/) and EPD (https://epd.epfl.ch/index.php) websites were used to predict the binding sites of FBXO5 and E2F2. The protein-protein interaction network of FBXO5 was generated using the BioGRID database (https://thebiogrid.org/).

### Cell culture and tissue samples

All cells used in the experimental study were purchased from the cell repository of the Shanghai Institute of Biological Sciences (Shanghai, China). The colon cancer cell line HCT116 was cultured in McCoy’s 5A medium (Procell) supplemented with reducing agent glutathione, trypticase peptone, and high glucose concentration. The SW480 cell line was cultured in IMDM medium (KeyGEN BioTECH). The HT-29 cell line was cultured in DMEM/F12 medium (KeyGEN BioTECH). All culture media were supplemented with 10% fetal bovine serum (FBS), 100 U/mL penicillin, and 100 mg/mL streptomycin. Endoplasmic reticulum stress induction was performed by adding 1 μM thapsigargin, 1 μM tunicamycin, 1 μM A23187 and 10 μM Brefeldin-A to cells at a certain confluency. 20 μM 4μ8C, 0.5 μM ISIRB and 10 μM Melatonin were used to specifically inhibit UPR receptors IRE1α, PERK and ATF6 respectively. Followed by subsequent treatments and corresponding detections after 12 h, cells were cultured in a humidified environment at 37 °C with 5% CO_2_. Throughout the experiment, measures were taken to ensure that all cells were free from mycoplasma contamination. Colon cancer tissue samples were obtained from the First People’s Hospital of Lianyungang. All samples were collected with informed consent from patients or subjects and their relatives and approved by the Medical Ethics Committee of the First People’s Hospital of Lianyungang.

### Cellular flow cytometric analysis

HCT116 cells were harvested and fixed at 4 °C overnight with 70% ethanol. According to the manufacturer’s protocol (KeyGEN BioTECH, Jiangsu, China), the fixed cells were incubated with RNase A and PI for 30–60 min at room temperature (25 °C) in the dark after washing and analyzed by flow cytometry (CytoFLEX, Beckman Coulter, CytExpert 2.4 version). ModFit LT 5.0 software (Verity Software House, Topsham, ME, USA) was used to analyze cell cycle distribution.

### Construction of stable cell lines

Plasmids expressing the lentiviral vectors, E2F2, RNF183, FBXO5, and FBXO5-ΔF-box were individually packaged with LentiPac plasmids. EndoFectin Lenti transfection reagent was added to form the DNA-EndoFectin transfection complex. The transfection complex was then evenly added dropwise to 293 T cell plates with a cell density ranging from 1.3 × 10^6^ to 1.5 × 10^6^. The cells were cultured at 37 °C with 5% CO_2_ for 48 h. Afterward, the cell culture medium was collected by centrifugation at 2000 × g for 10 min to remove cell debris, resulting in crude virus supernatant. Alternatively, cell debris was filtered using a 0.45 μm low-protein-binding PES filter membrane. Subsequently, a mixture of the crude virus and complete culture medium was prepared to infect the target HCT116 cells. The cells were selected with 2 μg/mL puromycin for 2 weeks to obtain stable expression cell lines.

### RNA interference, RNA isolation and real-time PCR

The lentiviral vectors for human RNF183 and FBXO5 were purchased from Merck (Sigma). The target sequences for the expression of short hairpin RNA (shRNA) plasmids are as follows: RNF183-shRNA: 5′-CCGGATCTTCGCCTATCTGAT-3′; E2F2-shRNA: 5′-GCCTATGTGACTTACCAGGAT-3′; FBXO5-shRNA1: 5′-TCGCTGTAATTCACCTGCAAA-3′; FBXO5-shRNA2: 5′-CGGATAGTTGTAAAGAAGAAA-3′. HCT116 cells were infected with the lentiviral plasmids (E2F2-shRNA; RNF183-shRNA, FBXO5-shRNA1, and FBXO5-shRNA2), followed by selection with 2 μg/mL puromycin for 2 weeks. Total RNA was isolated from the cultured human colon cancer cells using Trizol reagent (Invitrogen, USA). The isolated mRNA was reverse transcribed into cDNA using a reverse transcription kit (Promega). RT-PCR was performed on a QLightCycler480 instrument (Roche) with the following cycling conditions: 95 °C for 30 s; 95 °C for 5 s, 40 cycles; 62 °C for 30 s. Confirmed the specificity of the amplified products. GAPDH was used as an internal reference for normalization Ct values in each reaction. The primer sequences used were as follows: FBXO5 forward: 5′-GCTGTCATGTATTGGGTCACC-3′ and reverse: 5′-GTCTACTGGTCTCTAGTGCTTCT-3′; ATF4 forward: 5′-CTCCGGGACAGATTGGATGTT-3′ and reverse: 5′-GGCTGCTTATTAGTCTCCTGGAC-3′; sXBP1 forward: 5′-CCCTCCAGAACATCTCCCCAT-3′ and reverse: 5′-ACATGACTGGGTCCAAGTTGT-3′; CHOP forward: 5′-GGAAACAGAGTGGTCATTCCC-3′ and reverse: 5′-CTGCTTGAGCCGTTCATTCTC-3′; E2F2 forward: 5′-CGTCCCTGAGTTCCCAACC-3′ and reverse: 5′-GCGAAGTGTCATACCGAGTCTT-3′.

### Western blot analysis

After centrifugation of the cells, the culture medium was removed, and the cells were washed twice with PBS. Then, an appropriate amount of 1 × loading buffer (P0015A, Beyotime) was added to lyse the cells. Equal amounts of protein were separated using SDS-PAGE electrophoresis and transferred to a PVDF membrane. The membrane was blocked with 5% skim milk for 45 min. The membrane was then incubated overnight at 4 °C with the respective primary antibodies. After incubation with the primary antibodies, the membrane was placed on a shaker (75 rpm) and washed with PBST. Subsequently, the membrane was incubated with the respective secondary antibodies at room temperature for 45 min. The primary antibodies used were as follows: anti-FBXO5 (1:5000 dilution, PA5-83055, Thermo Fisher Scientific), anti-CHOP (1:500 dilution, sc-7351, Santa Cruz), anti-GAPDH (1:500 dilution, sc-47724, Santa Cruz), anti-Cleaved Caspase-3 (1:10,000 dilution, 9661, Cell Signaling Technology), anti-ATF4 (1:500 dilution, sc-390063, Santa Cruz), anti-P-eIF2α (1:10,000 dilution, 3998, Cell Signaling Technology), anti-eIF2α (1:10,000 dilution, 5324, Cell Signaling Technology), anti-ATF6 (1:10,000 dilution, 65880, Cell Signaling Technology), anti-sXBP1 (1:10,000 dilution, 12782, Cell Signaling Technology), anti-Bcl-xL (1:10,000 dilution, MA5-15142, Thermo Fisher Scientific), anti-β-actin (1:2000 dilution, AC004, Abclonal), anti-RNF183 (1:5000 dilution, PA5-63466, Thermo Fisher Scientific), anti-FLAG (1:10,000 dilution, 8146 S, Cell Signaling Technology), and anti-GST (1:500 dilution, sc-138, Santa Cruz).

### Immunoprecipitation (IP) and GST-Pull down

The cells stably expressing FBXO5 were lysed by rotating in an IP buffer (100 mM NaCl, 20 mM Tris-HCl, pH 8.0) containing 0.5 mM PMSF, 0.5 mM EDTA, and 0.5% (v/v) Nonidet P-40 at 4 °C for 1 h. Subsequently, the cells were sonicated on ice using a 5 s on/5 s off cycle for a total of 10 min of sonication. After completion, the lysate was centrifuged, and the supernatant was mixed with mouse IgG beads (P2171, Biyotime) and rotated at 4 °C for 4 h. After centrifugation, the supernatant was mixed with Flag-M2 beads (M8823, Sigma) and incubated overnight at 4 °C with rotation. The immunoprecipitated complexes were eluted with 3 × FLAG peptide (SRP0566, Sigma), and the eluates were subjected to immunoblot analysis. To examine the interaction between GST or GST-tagged RNF183 protein and the cell lysate expressing FLAG-FBXO5, the lysates were incubated overnight at 4 °C and then with glutathione beads for 30 min. The samples were processed with SDS buffer, boiled for 10 min, separated using SDS-PAGE, and subjected to immunoblot analysis.

### Chromatin Immunoprecipitation assay (ChIP)

The EZ-Magna ChIP TMA kit (Millipore, Billerica, MA) was used for ChIP analysis. Colorectal cancer cells in logarithmic growth phase were collected and incubated with 1% formaldehyde for 10 min. The reaction was stopped by adding 125 mM glycine for 5 min. The cells were resuspended in a cell lysis buffer (150 mM NaCl, 50 mM Tris pH 7.5, 5 mM EDTA, 0.005% NP40, 0.01% Triton X-100), followed by centrifugation at 5000 rpm for 5 min. After resuspension, the cells were sonicated, and then centrifuged. The supernatant was incubated overnight with either IgG or E2F2 antibody. The cleared lysates were mixed with 900 μL ChIP dilution buffer, 20 μL 50 × PIC, and 60 μL Protein A Agarose/Salmon, and incubated at 4°C for 2 h with rotation at 700 rpm. The supernatant was removed, and the pellet was washed twice with 1 mL each of low salt buffer, high salt buffer, LiCl solution, and TE buffer. Each tube was washed twice with 250 mL ChIP wash buffer, and the crosslinks were reversed by adding 20 mL 5 M sodium chloride, followed by DNA recovery. The enrichment of the FBXO5 promoter in the complexes was quantified using fluorescence quantitative PCR.

### Colony formation assay

HCT116 cells transfected with shRNA-RNF183, shRNA-FBXO5, or co-silenced for RNF183 and FBXO5 were seeded at a density of 1 × 10^3^ cells per well in a six-well plate and cultured for ~2 weeks. After fixation with 1% paraformaldehyde for 20 min, cells were stained with 0.05% crystal violet for 20 min, followed by gentle rinsing with running water. Bacterial colonies containing 50 or more cells were photographed and counted.

### Luciferase activity

To investigate the transcriptional regulation of FBXO5 by E2F2, 293 T cells were transfected with pGL4.15-FBXO5 WT or MUT and pGL4.15-Renilla (with or without E2F2). After transfection, cells were harvested and lysed, and luciferase activity was measured using the Dual-Luciferase Reporter Assay System (Promega, Madison, WI, USA). Luciferase activity was normalized to the activity of Renilla luciferase.

### BrdU assay

Cell proliferation was examined using the bromodeoxyuridine (BrdU) assay kit (Roche Applied Science, CA, USA). After transfection, cells were seeded in a 96-well plate at a density of 1 × 10^4^ cells per well and incubated for 48 h. When the cell confluence reached 80%, cells were labeled with BrdU for 24 h. Subsequently, cells were fixed for 30 min and washed twice with PBS. Permeabilization was achieved, and cells were incubated with BrdU antibody at room temperature for 2 h. Cell proliferation was measured using an enzyme-linked immunosorbent assay (ELISA) reader at a wavelength of 450 nm.

### Apoptosis assay

Cultured cells from different treatments were collected and analyzed for apoptotic cells using the FITC Annexin V Apoptosis Detection Kit I (BD Biosciences). Approximately 1 × 10^6^ cells were suspended in 1 × binding buffer and incubated with membrane-associated protein V-FITC and propidium iodide (PI) at room temperature (25 °C) for 15 min. The mixture was then analyzed using a flow cytometer, and data analysis was performed using FlowJo version 10.8.1 software (Tree Star). The level of cell apoptosis was detected using the APOAF Annexin V-FITC Apoptosis Detection Kit (Sigma). The stained cells were then analyzed using a FACSCalibur flow cytometer (BD Biosciences), and the collected data were analyzed using FlowJo software. Forward scatter and side scatter were used for gating to exclude any cell debris. Ninety microliters of HCT116 cells (2000 cells) were seeded in a 96-well plate and incubated overnight. The cells were then induced to undergo apoptosis for a certain period of time in a CO_2_ incubator at 37 °C. Following this, 100 μL of caspase 3/7 detection buffer was added to each well, and the plate was incubated at room temperature in the dark for 1 h before measuring the fluorescence intensity.

### Xenograft tumors

BALB/c nude mice were purchased from the National Rodent Laboratory Animal Resources Company (Shanghai, China) and housed in specific facilities. The mice were randomly divided into two groups, with five mice in each group. A suspension of HCT116 cells (1 × 10^7^ cells) expressing shRNA-FBXO5 or a control (sh-Con) in 50 μL of DMEM medium was prepared and injected subcutaneously into the corresponding groups of mice. After 4 weeks, the tumors were excised, and the tumor weight was measured. Photographs were taken. In another experiment, the mice were randomly divided into groups of five. HCT116 cells (1 × 10^7^ cells) transfected with sh-FBXO5 and sh-RNF183 alone or simultaneously were injected subcutaneously on both sides of the mouse The tumor growth was observed regularly for 4 weeks, and the tumor volume and growth rate were measured. Photographs were taken. This animal study was approved by the Ethics Committee of Jiangsu Ocean University.

### Immunohistochemistry (IHC)

Transplanted tissue samples from mice were fixed in 10% formalin buffer, embedded in paraffin, and sliced at a thickness of 5 μm. The slices were then dried, deparaffinized, rehydrated, and subjected to antigen retrieval by boiling in citrate buffer (pH 6.0). The slices were incubated overnight at 4 °C with anti-FBXO5 (diluted 1:100, PA5-83055, Thermo Fisher Scientific) and anti-CHOP (diluted 1:100, sc-7351, Santa Cruz). Anti-rabbit secondary antibodies from an immunohistochemistry staining kit (ZSGB-BIO, Beijing, China) were added to each sample for 30 min, followed by visualization with 3,3′-diaminobenzidine (DAB, ZSGB-BIO, Beijing, China). Slices were stained with hematoxylin and eosin (H&E) for light microscopic evaluation. IHC staining was analyzed and quantified by two independent pathologists using a double-blind method.

### Molecular docking method

The proteins FBXO5 and RNF183 were expertly docked using molecular docking software programs ClusPro2 and RosettaDock4 [16]. Due to the unavailability of resolved three-dimensional structures for FBXO5 and RNF183, their predicted conformations (UniProt: Q9UKT4 and Q96D59, respectively) were initially acquired from the Alphafold2 database (https://alphafold.ebi.ac.uk/). Subsequently, a comprehensive docking search was performed using the ClusPro2 platform (https://cluspro.org/dimer_predict/submit.php) [17]. In order to optimize the identification of interactions between FBXO5 and RNF183, their interface was left undefined before docking. During the procedure, ClusPro2 utilized a fast Fourier transform (FFT)-based algorithm for rigid body docking. Four unique clusters were generated, and the structure with the lowest energy in each cluster was selected for precise local docking. RosettaDock4 (https://r2.graylab.jhu.edu/apps/submit/docking) [18] enabled this refined docking, and the conformation with the lowest overall energy among the four results was considered ideal for further structural analysis.

### Statistical analyses

Statistical analysis was conducted using GraphPad Prism 9.0 software (USA, San Diego, CA). Differences between two groups were assessed using the Student’s *t*-test or one-way analysis of variance (ANOVA) followed by Tukey’s test. Data are presented as mean ± standard deviation (SD). Statistical significance was represented as *p < 0.05, **p < 0.01*, and* ***p < 0.001.

## Results

### ER stress suppresses E2F target genes expression and induces G2/M checkpoint arrest in colon cancer cells

**Fig. 1:**
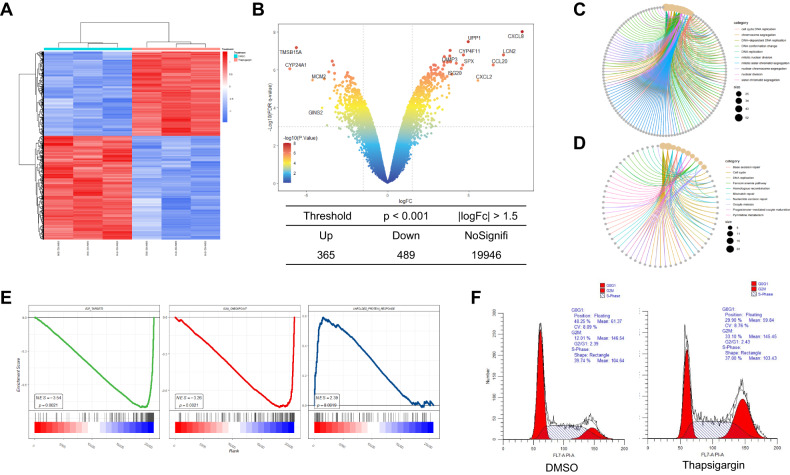
ER stress suppresses E2F target genes expression and induces G2/M checkpoint arrest in colon cancer cells. **A** The GSE41666 dataset contained microarray data from DMSO or thapsigargin treated HCT116 cells was normalized by limma package and heatmap of clustering analysis was performed ( | log2 fold change | > 1.5 and *p* < 0.001). **B** The volcano plot displaying the fold changes of genes caused by thapsigargin treatment. The vertical and horizontal dashed lines indicate log2 fold change and *p*-value cutoff, respectively. Red dots represent upregulated genes and blue dots represent downregulated gene. The figure below shows the total number of different expressed genes. **C** GO analysis of the significantly downregulated genes for biological process (BP), cellular compartment (CC), and molecular function (MF). **D** KEGG pathway analysis of the significantly downregulated genes. **E** Relative biological functions of different expressed genes were verified by GSEA analyses. **F** Cell cycle analysis of HCT116 in DMSO or thapsigargin-treated groups by flow cytometry.

To uncover the modulators involved in the progression of endoplasmic reticulum (ER) stress, we compared the expression profiles of HCT116 colon cancer cells that were treated with either DMSO or thapsigargin using data from GSE41666 (Fig. 1A). Using a threshold of *p* < 0.001 and |log2 fold change | > 1.5, we narrowed down the candidate genes. The results revealed 365 upregulated genes and 489 specifically downregulated genes in response to thapsigargin treatment (Fig. 1B, Supplementary Tables 1, 2). Functional enrichment analysis showed that the upregulated genes were enriched in processes related to ER stress and unfolded protein response, as well as the ubiquitin-dependent ER-associated degradation pathway (Supplementary Fig. 1A, B), suggesting that the thapsigargin treatment was effective in activating the ER response.

Of particular interest were the downregulated genes, which were significantly enriched in biological processes related to the cell cycle, DNA replication, DNA repair, and spindle (Fig. 1C, D). Further analysis using Gene Set Enrichment Analysis (GSEA) indicated that the hallmark gene set for the unfolded protein response was upregulated in the thapsigargin-treated group (Fig. 1E), while the hallmark gene sets for the G2/M checkpoint and E2F targets were downregulated. In addition, the results of the cell cycle by flow cytometry also showed that the cell cycle was significantly blocked in the G2/M phase in the thapsigargin-treated group (Fig. 1F). These findings suggest that ER stress upregulates the UPR signaling pathway while simultaneously inhibiting the expression of E2F target genes and downregulating the progress of the G2/M phase in colon cancer cells.

### ER stress selectively down-regulates FBXO5 expression

**Fig. 2:**
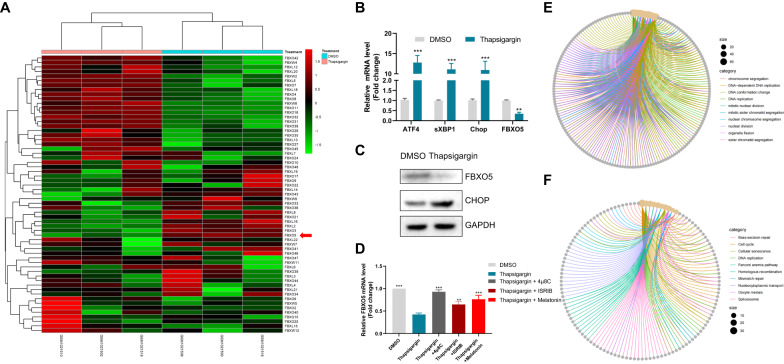
ER stress selectively down-regulates FBXO5 expression. **A** Heatmap of clustering analysis showed the expression of F-box family members (with 64 quantifiable members) following thapsigargin treatment. **B** Relative mRNA fold change of ATF4, sXBP1, CHOP, and FBXO5 in HCT116 cells treated with 1 μM thapsigargin for 12 h. **p < *0.01 vs* Con, ***p < *0.001 vs* Con. **C** Western blot analysis the level of FBXO5 and CHOP in HCT116 cells treated with 1 μM thapsigargin for 12 hr. **D** Relative mRNA fold change of FBXO5 in HCT116 cells treated with DMSO, thapsigargin (1 μM) with or without 4μ8C (20 μM), ISRIB (0.5 μM) and Melatonin (10 μM). **p < *0.01 vs* thapsigargin, ***p < *0.001 vs* thapsigargin. **E** GO analysis of 200 co-expressed genes of FBXO5 from TCGA COAD tumor dataset for biological process (BP), cellular compartment (CC), and molecular function (MF). **F** KEGG pathway analysis of 200 co-expressed genes of FBXO5 from TCGA COAD tumor dataset.

Since F-box family proteins play a fundamental role in cell cycle control, the expression of all F-box family members was tested in HCT116 cells following treatment with thapsigargin. Out of 61 quantifiable F-box family proteins, significant downregulation of FBXO5 and FBXL2, and significant upregulation of FBXO16 and FBXO32 were observed (Fig. 2A, Supplementary Table 3). No notable alterations were detected among the remaining F-box family members. Given the known role of FBXL2 as a tumor suppressor gene in various tumors, we focused on FBXO5. Real-time PCR results showed that while treatment with thapsigargin induced expression of well-known ER stress markers, ATF4, sXBP1, and CHOP, it also significantly downregulated FBXO5 expression (Fig. 2B). Western blotting confirmed that the protein level of FBXO5 was decreased upon thapsigargin treatment, contrasting the expression of CHOP (Fig. 2C). The validity of these findings was further reinforced by the results showing that three additional UPR inducers, specifically tunicamycin, A23187, and Brefeldin-A, led to significant inhibition of FBXO5 and E2F2 expression in multiple colon cancer cell lines, including HT29, HCT116, and SW480 (Supplementary Fig. 2A–G). Alleviation of ER stress by specific inhibitors of the three proximal sensors of UPR, namely 4μ8C, ISRIB, and Melatonin, attenuated the downregulation of FBXO5 induced by thapsigargin in HCT116 cells (Fig. 2D). These results demonstrate a link between activation of ER stress and FBXO5 down regulation in colon cancer cells. Interestingly, GO analysis of 200 co-expressed genes of FBXO5 in TCGA colon tumor tissues revealed a relationship between FBXO5 and cell cycle, DNA replication, DNA repair, and spindle pathways, all of which are down regulated by ER stress (Fig. 2E, F, Supplementary Table 4). These results suggest that ER stress-induced down regulation of FBXO5 might be associated with ER stress-mediated cell cycle inhibition.

### ER stress-induced E2F2 downregulation is required for FBXO5 repression

**Fig. 3:**
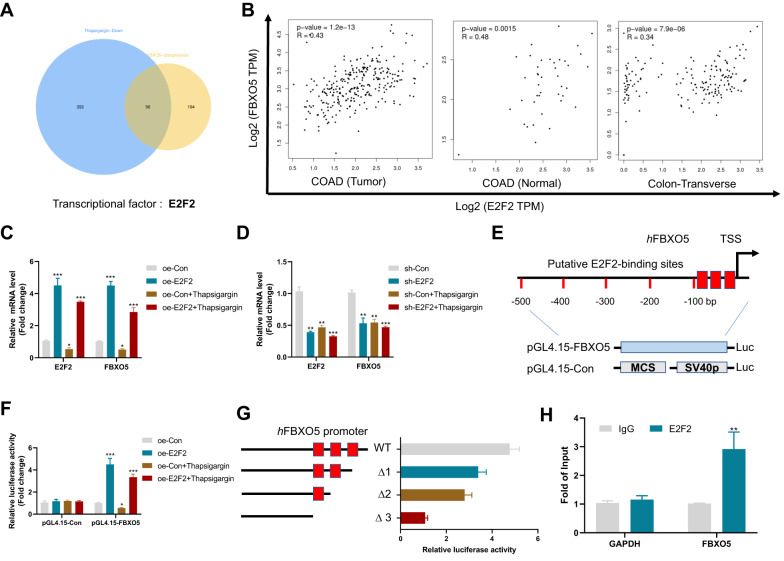
ER stress-induced E2F2 downregulation is required for FBXO5 repression. **A** The top 200 co-expressed genes of FBXO5 in TCGA COAD tumor dataset and 489 downregulated genes following thapsigargin treatment were analyzed by Venn diagram, and there were 96 common genes in the two data sets. **B** The correlation of the mRNAs of FBXO5 and E2F2 in GEPIA2 website including the COAD tumor, COAD normal from TCGA database and the normal colon tissues (Colon-Transverse) from the GTEx database. **C** Relative mRNA fold changes of FBXO5 and E2F2 after transfection of vector control or E2F2 in HCT116 cells treated with DMSO or thapsigargin. *p < *0.05 vs* oe-Con, ***p < *0.001 vs* oe-Con. **D** Relative mRNA fold changes of FBXO5 and E2F2 after transfection of control sh-RNA or E2F2 sh-RNA in HCT116 cells treated with DMSO or thapsigargin. **p < *0.01 vs* sh-Con, ***p < *0.001 vs* sh-Con. **E** Schematic diagram shows FBXO5 gene promoter and three putative E2F2 binding sites. TSS, transcription start site. **F** The pGL4.15-Con or pGL4.15-E2F2 plasmids were transfected with vector control or E2F2, respectively, into 293 T cells, which were subsequently treated with DMSO or thapsigargin for 12 h. The luciferase activity was then measured. *p < *0.05 vs* oe-Con, ***p < *0.001 vs* oe-Con. **G** The FBXO5 gene promoter contains three potential binding sites for E2F2. The transcriptional activity of wild-type or mutant FBXO5 promoters in 293 T cells transfected with E2F2 was determined. H. Vector control and E2F2 were cotransfected into HCT116 cells and CHIP experiments were performed. **p < *0.01 vs* IgG.

To understand the mechanism behind FBXO5 down regulation in response to ER stress, we performed a comparative analysis of the 200 co-expressed proteins of FBXO5 in colon cancer and the 489 downregulated proteins in HCT116 cells after thapsigargin treatment. Of the 96 proteins that belong to both datasets, only one transcriptional factor, E2F2, was identified (Fig. 3A, Supplementary Tables 4, 5). Notably, among the 8 members of the E2F family, thapsigargin treatment selectively downregulated E2F2 and E2F8 (Supplementary Fig. 2H). Given that the main biological function of E2F8 is to repress the expression of E2F-regulated genes, we focused on the relationship between E2F2 and FBXO5. Analysis of RNA-seq data from colon cancer (COAD cancer) and non-colon cancer tissues (COAD normal) from the TCGA database and normal colon tissues (Colon-Transverse) from the GTEx database revealed a strong correlation between the expression of E2F2 and FBXO5 (Fig. 3B), suggesting that FBXO5 may be regulated by E2F2. Consistent with this, overexpression of E2F2 in HCT116 cells significantly upregulated the expression of FBXO5, and this upregulation was still effective after thapsigargin addition (Fig. 3C). Knockdown of E2F2 resulted in a decrease in FBXO5 expression, as well as a weakened ability of thapsigargin to inhibit FBXO5 (Fig. 3D). A search of the Eukaryotic Promoter Database (EPD) revealed that the promoter region of FBXO5 contains three putative E2F2 binding sites located from −100 to 0 bp relative to the transcription start site (TSS) (Fig. 3E). Luciferase reporter assays using a vector under the control of the E2F2 promoter, containing all three putative elements (−1000 to +1 bp of TSS), showed that E2F2 could induce 4-6-fold activation of the FBXO5 promoter luciferase activity in the presence or absence of thapsigargin (Fig. 3F). Deleting one or two of these sites partially inhibited the E2F2-driven FBXO5 promoter luciferase activity, while deletion of all three elements almost completely abolished it (Fig. 3G). Chromatin immunoprecipitation (ChIP) assays confirmed abundant E2F2 occupation at the FBXO5 promoter (Fig. 3H). These results demonstrate that E2F2 is required for FBXO5 repression in response to ER stress.

### FBXO5 prevents ER stress-induced apoptosis of colon cancer cells by inhibiting the activation of IRE1α, ATF6 and PERK signaling pathways

**Fig. 4:**
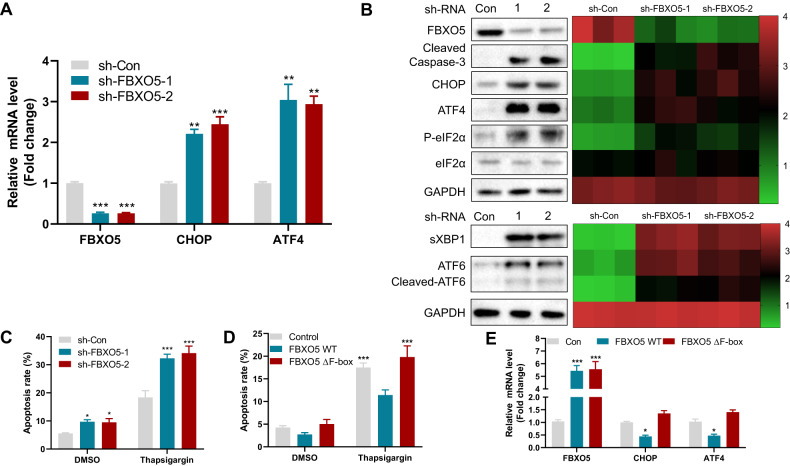
FBXO5 prevents ER stress-induced apoptosis of colon cancer cells by inhibiting the activation of PERK/IRE1α/ATF6 signaling pathway. **A** Relative mRNA fold change of FBXO5, CHOP and ATF4 in HCT116 cells after stable knockdown of FBXO5 by two different shRNAs. **p < 0.01 *vs* sh-Con, ***p < 0.001 *vs* sh-Con. **B** Western blot analysis of HCT116 cells after stable knockdown of FBXO5 by two different shRNAs with indicated antibodies. The quantitative information of each protein of the triplicate experiments was obtained by Image J software and displayed by the heatmap on the right side. **C** Quantification of apoptotic HCT116 cells stable knockdown of FBXO5 with or without 1 μM thapsigargin treatment for 12 h and analyzed by flow cytometry. *p < *0.05 vs* sh-Con, ***p < 0.001 *vs* sh-Con. **D** Quantification of apoptotic HCT116 cells overexpression of FBXO5 WT or FBXO5-∆F-box mutant, with or without 1 μM thapsigargin treatment for 12 hr and analyzed by flow cytometry. ***p < *0.001 vs* FBXO5 WT. **E** Relative mRNA fold change of CHOP and ATF4 in HCT116 transfected with FBXO5 WT or FBXO5-∆F-box mutant. *p < *0.05 vs* Con, ***p < *0.001 vs* Con.

FBXO5 is known to play a crucial role in the regulation of the cell cycle, particularly at the G2/M checkpoint, by suppressing the activity of the APC/C E3 ligase. However, its involvement in the response to endoplasmic reticulum (ER) stress remained unclear. To address this, we investigated the role of FBXO5 in ER stress-induced apoptosis. We employed two different shRNAs to downregulate FBXO5 expression and observed that both shRNAs reduced FBXO5 expression by 70–80% in cells (Fig. 4A). This resulted in decreased proliferation of HCT116 and SW480 cells (Supplementary Fig. 3A, B). Furthermore, FBXO5 knockdown led to elevated expression of cleaved caspase-3 (Fig. 4B), and increased thapsigargin-induced apoptosis in HCT116 cells (Fig. 4C). Conversely, overexpression of FBXO5, but not the FBXO5-ΔF-box mutant, counteracted thapsigargin-induced apoptosis (Fig. 4D), suggesting that FBXO5’s inhibitory effect on thapsigargin-induced apoptosis is linked to its E3 ligase activity. Similar results were obtained when HCT116 cells were treated with tunicamycin and subjected to either FBXO5 knockdown or overexpression (Supplementary Fig. 4A), suggesting that FBXO5 plays a role in ER stress-induced apoptosis in colon cancer cells.

To explore the molecular mechanism by which FBXO5 regulates ER stress-induced apoptosis, we found that FBXO5 depletion significantly increased the genes expression involved in the IRE1α, ATF6 and PERK signaling pathways, including spliced XBP1, cleaved-ATF6, phosphorylated eIF2α, CHOP and ATF4 (Fig. 4A, B). These three signaling pathways are the major pathways associated with ER stress. Conversely, overexpression of FBXO5, but not the FBXO5-ΔF-box mutant, reduced CHOP and ATF4 expression (Fig. 4E). Collectively, these findings suggest that FBXO5 may regulate ER stress by targeting an unknown upstream factor of IRE1α, ATF6 and PERK signaling pathways for degradation.

### Knockdown of FBXO5 suppresses colon cancer growth in nude mice

**Fig. 5:**
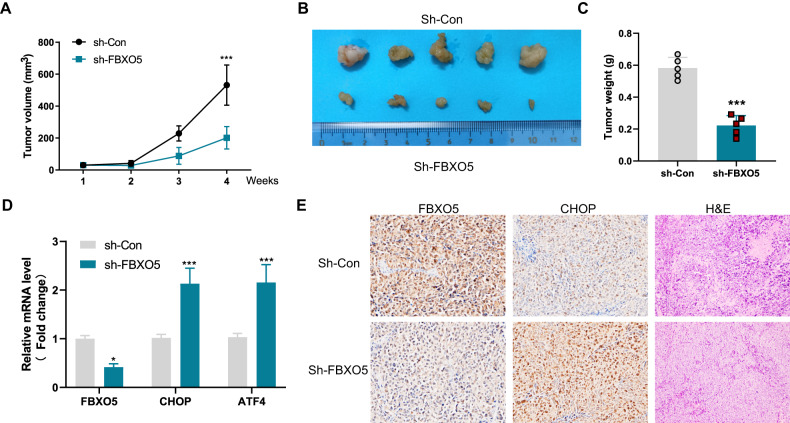
Knockdown of FBXO5 suppresses colon cancer growth in nude mice. **A** HCT116 cells stably expressing sh-Control or sh-FBXO5 were subcutaneously injected in nude mice respectively. Shown are average tumor volumes over time (*n* = 5). ***p < *0.001 vs* sh-Con. **B** The representative tumor image at day 28**. C** The weights of tumors at day 28. ***p < *0.001 vs* sh-Con. **D** Relative mRNA fold change of FBXO5, CHOP and ATF4 in tumor derived from HCT116 cells stably expressing sh-Con or sh-FBXO5. *p < *0.05 vs* sh-Con. ***p < *0.001 vs* sh-Con. **E** Representative IHC and H&E staining images of FBXO5 and CHOP in tumor derived from HCT116 cells stably expressing sh-Con or sh-FBXO5.

As ER-mediated FBXO5 downregulation plays an important role in ER-induced colon cancer cell apoptosis, we then investigated whether FBXO5 is involved in the oncogenesis of colon cancer. To examine the role of FBXO5 in vivo, we explanted HCT116 cells with control or FBXO5 knockdown in nude mice. Compared with the control knockdown group, the FBXO5 knockdown group displayed significantly smaller tumor volume and lighter tumor weight in nude mice (Fig. 5A–C). Through real-time PCR and IHC analysis, we found that FBXO5 knockdown tumor tissues showed increased CHOP expression (Fig. 5D, E). Together, these results demonstrate that FBXO5 promotes colon cancer progression in vivo through modulating the expression of CHOP.

### Upregulation of FBXO5 in colon cancer

**Fig. 6:**
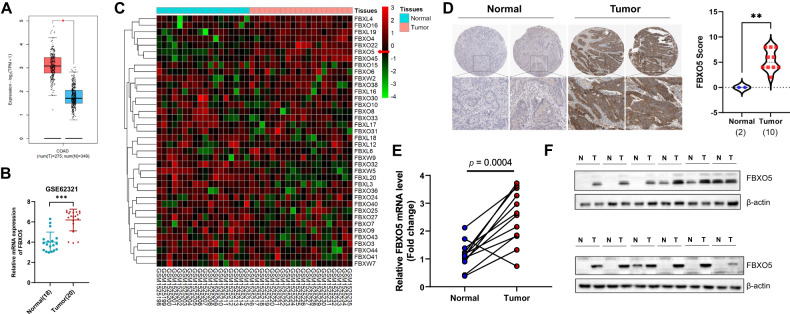
Upregulation of FBXO5 in colon cancer. **A** FBXO5 mRNA expression of tumor and normal tissues in TCGA Colon adenocarcinoma (COAD). *p < 0.05 *vs* normal. **B** FBXO5 mRNA expression of colon tumor and normal colon tissues in GSE62321 microarray dataset (Normal = 18, Tumor = 20). ***p < 0.001 *vs* normal. **C** The mRNA levels of 40 F-box protein family members of colon tumor and normal colon tissues in GSE62321 microarray dataset (Normal = 18, Tumor = 20). **D** Representative IHC staining images of colon tumor and adjacent normal colon tissues. **p < 0.01 *vs* normal. **E** FBXO5 mRNA expression of 12 colon tumor and adjacent normal colon tissues. F. FBXO5 protein expression of 12 colon tumor and adjacent normal colon tissues.

To unravel the clinical relevance of FBXO5 in cancer, we further explored the expression of FBXO5 in clinical colorectal cancer tissues. We observed elevated expression of FBXO5 mRNA in COAD tumor tissues in both the TCGA database and the GSE62321 colorectal cancer patient cohort (Fig. 6A, B). Importantly, in the GSE62321 cohort, among the 38 quantifiable F-box proteins, FBXO5 was the only significantly upregulated F-box protein in colorectal cancer tissues (Fig. 6C, Supplementary Table 6), further indicating its key role in colorectal cancer. Additionally, analysis of the human protein atlas database revealed an elevated level of FBXO5 protein in colorectal cancer tissues compared to normal samples, as confirmed by immunohistochemical (IHC) staining using anti-FBXO5 antibodies (Fig. 6D). Furthermore, validation in clinical colorectal cancer tissues using real-time PCR and western blot corroborated the higher levels of FBXO5 in tumor tissues compared to adjacent normal tissues (Fig. 6E, F). Taken together, these finding demonstrates a noteworthy upregulation of FBXO5 in human colorectal cancer (COAD), highlighting its potential as a therapeutic target for cancer prevention.

### FBXO5 regulates the stability and ubiquitination of RNF183

**Fig. 7:**
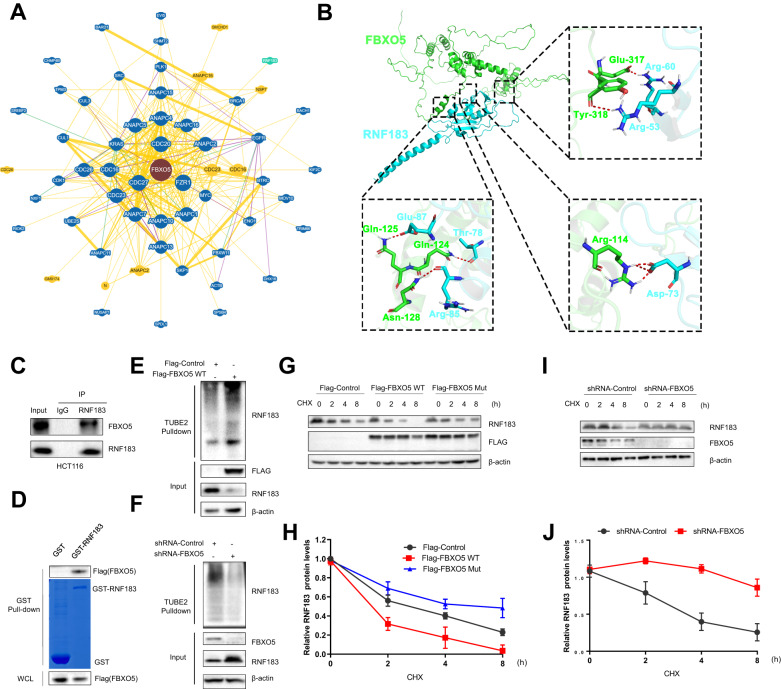
FBXO5 regulates the stability and ubiquitination of RNF183. **A** Protein interaction network of FBXO5 shown in the BioGRID database (https://thebiogrid.org/). **B** Interaction between FBXO5 (green) and RNF183 (blue) analyzed using the PLIP software. **C** IgG and anti-RNF183 binding magnetic beads, the mixed magnetic beads were added to HCT116 cells, followed by immunoblotting with the indicated antibodies. **D** In vitro GST pull-down assay for the interaction of RNF183 in HCT116 cells. **E** Immunoprecipitation using TUBE2 resin of cell lysates after transfection of Flag-Con or Flag-FBXO5 plasmids in HCT116 cells for 36 h. The precipitated proteins were analyzed by immunoblotting. **F** Immunoprecipitation using TUBE2 resin of cell lysates after transfection of sh-Con or sh-FBXO5. **G** Immunoblot analysis of RNF183 in HCT116 cells transfected with Control, FBXO5 WT or FBXO5 Mut treated with Cycloheximide (CHX) (25 μg/mL) for the indicated durations. **H** Quantification of relative RNF183 protein levels in (**G**). **I** Immunoblot analysis of RNF183 in HCT116 cells transfected with sh-control and sh-FBXO5 treated with Cycloheximide (CHX) (25 μg/mL) for the indicated durations. **J** Quantification of relative RNF183 protein levels in (**I**).

To investigate the molecular mechanism by which FBXO5 regulates cancer progression, we searched the BioGRID database (https://thebiogrid.org/) for protein interactions of FBXO5. There were 57 proteins identified to interact with FBXO5 (Fig. 7A). Deletion of FBXO5 resulted in increased apoptotic cell death induced by endoplasmic reticulum stress. Interestingly, we found that RNF183 was listed as one of the interacting proteins with FBXO5. RNF183 is a member of the RING E3 family, located in the endoplasmic reticulum. During sustained endoplasmic reticulum stress, the protein level of RNF183 increase, and it interacts with the anti-apoptotic protein Bcl-xL, leading to its degradation and the initiation of cell apoptosis [19]. This prompted us to focus on the relationship between FBXO5 and RNF183. Next, we analyzed the binding interface of the FBXO5-RNF183 protein complex using the PLIP software. The results showed that the main residues involved in the interaction were Arg-114, Asn-120, Gln-124, Gln-125, Leu-127, Asn-128, Glu-317, Tyr-318 of FBXO5, and Arg-53, Leu-55, Arg-60, Asp-73, Thr-78, Leu-81, Ala-82, Arg-85, Glu-87 and Pro-88 of RNF183 (Fig. 7B). The interaction between FBXO5 and RNF183 was confirmed by endogenous immunoprecipitation (co-IP) in HCT116 cells (Fig. 7C). GST pull-down experiments further validated the direct binding of RNF183 with FBXO5 in vitro (Fig. 7D). Therefore, these results indicate an interaction between FBXO5 and RNF183. Further, we analyzed the regulation of RNF183 protein half-life and ubiquitination status by FBXO5. Compared to the control group, overexpression of FBXO5 increased the ubiquitination of RNF183 (Fig. 7E). Conversely, silencing of FBXO5 decreased its ubiquitination (Fig. 7F). Additionally, overexpression of FBXO5 significantly reduced the half-life of RNF183 protein (Fig. 7G, H), while silencing of FBXO5 significantly inhibited the degradation of RNF183 protein (Fig. 7I, J). These findings imply that FBXO5 has the ability to bind to RNF183 and regulate its ubiquitination and stability.

### FBXO5-mediated promotion of colon cancer development through RNF183

**Fig. 8:**
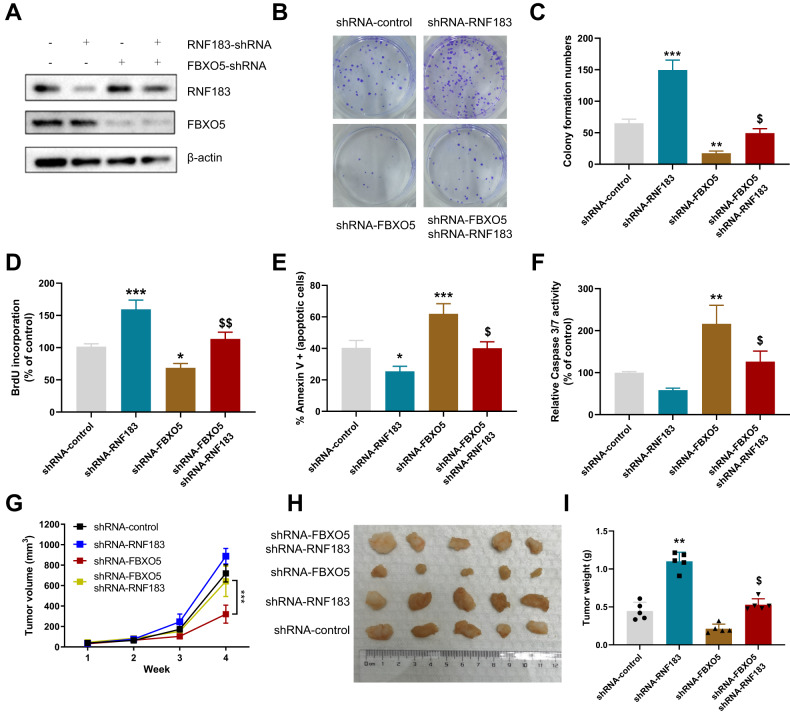
FBXO5 promotes tumor development through RNF183. **A** Transfection of shRNA-Control, shRNA-RNF183 and shRNA-FBXO5 in HCT116 cells followed by Western blot analysis to measure the levels of FBXO5 and RNF183. **B** Assessment of the effect of the four groups (Control, sh-RNF183, sh-FBXO5, and co-silencing) on colony formation in HCT116 cells. **C** Conducting counting and statistical analysis on (**B**). **p < 0.01 *vs* shRNA-Control, ***p < 0.001 *vs* shRNA-Control, ^$^*p* < 0.05 *vs* shRNA-FBXO5. **D** Evaluation of the growth capabilities of the four HCT116 cell types, representative BrdU staining and quantitative analysis of proliferating cells. ***p < 0.001 *vs* shRNA-Control, ^$$^*p* < 0.01 *vs* shRNA-FBXO5. **E** Assessment of the apoptotic levels in the four HCT116 cell lines. *p < 0.05 *vs* shRNA-Control, ***p < 0.001 *vs* shRNA-Control, ^$^*p* < 0.05 *vs* shRNA-FBXO5. **F** Assessment of changes in the apoptotic marker Caspase3/7 in the four HCT116 cell lines. **p < 0.01 *vs* shRNA-Control, ^*$*^*p* < 0.05 *vs* shRNA-FBXO5. **G** Subcutaneous injection of the four HCT116 cell lines (5 × 10^6^ cells per mouse) into 4-week-old BALB/c nude mice. Shown are average tumor volumes over time (*n* = 5). ***p < 0.001 *vs* shRNA-FBXO5. **H** The representative tumor image at day 28. **I** The weights of tumors at day 28. **p < 0.01 *vs* shRNA-Control, ^$^*p* < 0.05 *vs* shRNA-FBXO5.

**Fig. 9:**
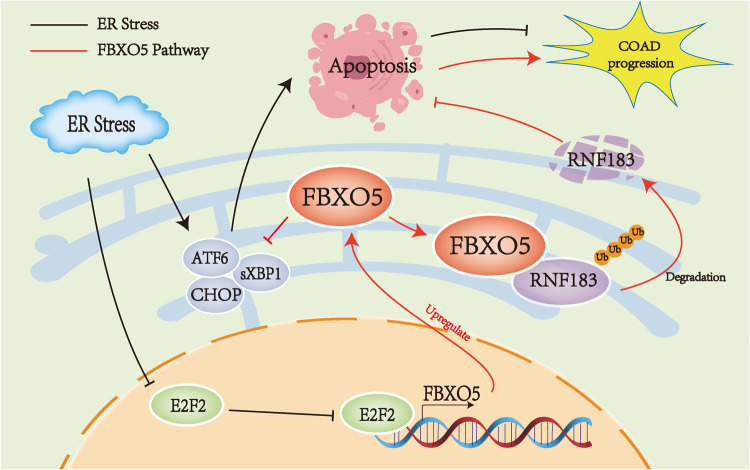
Working model. In this working model, we observed that ER stress inhibits E2F2-mediated expression of FBXO5. Our investigation revealed that FBXO5 is overexpressed in colorectal cancer, where it prevents ER stress-induced cell apoptosis by ubiquitinating and degrading RNF183, thereby promoting the progression of colorectal cancer.

After demonstrating the ability of FBXO5 to target and degrade RNF183, we further investigated whether FBXO5 relies on RNF183 to exert its anti-apoptotic effects. We employed transfections of shRNA-RNF183, shRNA-FBXO5, or a combination of both in HCT116 cells to silence RNF183, FBXO5, or both simultaneously (Fig. 8A). The results revealed that silencing FBXO5 resulted in a decelerated growth of colon cancer cells, while silencing RNF183 effectively reversed the growth arrest induced by FBXO5 silencing (Fig. 8B, C). Furthermore, silencing RNF183 enhanced the proliferative capacity of tumor cells and restored the proliferation ability of cells following FBXO5 silencing (Fig. 8D). Subsequently, we monitored apoptotic markers and found that with FBXO5 silencing, increased expression of RNF183 led to augmented Caspase3/7 activity, leading tumor cells towards apoptosis. Silencing RNF183 in FBXO5-silenced HCT116 cells was able to reverse the apoptosis induced by FBXO5 (Fig. 8E, F). Next, we explored the potential in vivo functions of FBXO5 by subcutaneously implanting control cells and three stably silenced HCT116 cell lines into 4-week-old BALB/c nude mice. After 4 weeks of observation, tumor size indicated that FBXO5 silencing suppressed tumor occurrence. Furthermore, when RNF183 was silenced in conjunction with FBXO5, tumor growth was restored, as evidenced by consistent conclusions drawn from statistical analyses of tumor volume and weight (Fig. 8G–I). Additionally, we investigated the impact of FBXO5 on RNF183 and the anti-apoptotic protein Bcl-xL during ER stress induced by thapsigargin treatment. The results demonstrated that silencing FBXO5 upregulated the protein expression of RNF183, irrespective of the presence or absence of thapsigargin. Conversely, there was a significant decrease in Bcl-xL protein expression (Supplementary Fig. 4B). Taken together, the results from both in vitro *and* in vivo studies suggest that FBXO5 exerts its anti-apoptotic effects and promotes tumor growth through the mechanism of targeted degradation of RNF183 Fig. 9.

## Discussion

Persistent ER stress induces UPR and can lead to apoptosis in colorectal cancer [20], but the exact molecular mechanisms are still unknown despite ongoing research. Against this background, the present study demonstrates that ER stress can suppress the expression of FBXO5 by inhibiting E2F2-mediated transcription. Secondly, FBXO5 is capable of suppressing ER stress-induced apoptosis in colon cancer cells. Thirdly, we discovered a significant increase in FBXO5 expression in human colorectal cancer. Furthermore, we demonstrated that FBXO5 plays a crucial role in the progression of colon cancer by promoting the ubiquitination and degradation of RNF183, which leads to the inhibition of ER stress-induced cell apoptosis.

The E2Fs are a family of DNA binding proteins that play a crucial role in regulating cell cycle progression by controlling the expression of genes involved in cell cycle regulation [21, 22]. In our study, we observed that ER stress not only activates the unfolded protein response (UPR) signaling pathway but also significantly down-regulates the expression of E2F target genes, thereby inhibiting the G2/M transition. Interestingly, the ER stress inducer thapsigargin specifically reduced the expression of E2F2 and E2F8. Since the E2F family members have critical and overlapping functions [23], it is reasonable to speculate that the downregulation of E2F2 may also be essential for ER stress-induced apoptosis. Considering the pivotal role of SCF E3 complexes in cell cycle control, we investigated whether ER stress could affect the activity of certain SCF complexes [24]. We discovered that FBXO5, a member of the F-box protein family, was one of the most significantly suppressed genes in response to ER stress, and its expression was primarily regulated by E2F2. Intriguingly, gene ontology analysis of FBXO5 co-expressed genes in colon tumor tissues revealed that FBXO5 is associated with cell cycle progression, DNA replication, DNA repair, and spindle pathways, all of which are downregulated during ER stress. This suggests that the selective downregulation of FBXO5 by ER stress may play a crucial role in ER stress-induced cellular processes. Functional investigation further demonstrated that FBXO5 promotes the survival of colon cancer cells by inhibiting UPR signaling pathways, leading to decreased ER stress and diminished pro-apoptotic activity. These findings provide valuable insights into the intricate molecular mechanisms underlying ER stress-induced cellular responses, specifically the involvement of E2F2 and FBXO5 in the regulation of cell cycle progression and apoptosis in colon cancer cells.

Furthermore, FBXO5 is significantly upregulated in colon cancer tissues, and knockdown of FBXO5 suppresses tumorigenesis in in vivo nude mice models, indicating that increased FBXO5 expression may contribute to colon cancer tumorigenesis by inhibiting ER stress-induced apoptosis. Interestingly, a novel interaction between FBXO5 and RNF183, an E3 ligase, has been discovered. RNF183 is primarily localized in the endoplasmic reticulum (ER) and plays a crucial regulatory role in cell survival and apoptosis, ultimately impacting tumor development [19, 25, 26]. Under prolonged ER stress, RNF183 protein levels are elevated, leading to its interaction with the anti-apoptotic protein Bcl-xL [19]. This interaction results in the degradation of Bcl-xL and the initiation of apoptosis, significantly influencing tumor suppression [27]. Moreover, the study demonstrates that FBXO5 overexpression enhances the ubiquitination modification of RNF183, resulting in a marked decrease in the half-life of RNF183 protein. By mediating ubiquitination-mediated degradation of RNF183, FBXO5 prevents ER stress-induced apoptosis and facilitates tumor growth.

This study is the first to discover that FBXO5 acts as a novel negative regulator of ER stress via E2F2. Additionally, FBXO5 is found to be upregulated in colon cancer and functions by inhibiting ER stress-induced apoptosis through promoting the ubiquitination degradation of RNF183, thereby contributing to colon cancer progression. However, many questions remain unanswered, including the specific region where FBXO5 binds to RNF183 and the type of ubiquitination degradation mediated by FBXO5 on RNF183. In conclusion, our research highlights FBXO5 as a potential therapeutic target for colon cancer treatment. The FBXO5-RNF183 axis shows promise for clinical cancer research and requires further exploration in this field.

### Supplementary information

supplementary data

Supplementary Table1

Supplementary Table2

Supplementary Table3

Supplementary Table4

Supplementary Table5

Supplementary Table6

Supplementary Figure legend

Reproducibility checklist

## Data Availability

The experimental data sets generated and/or analyzed during the current study are available from the corresponding author upon reasonable request. No applicable resources were generated during the current study.

## References

[1] Oakes SA, Papa FR (2015). The role of endoplasmic reticulum stress in human pathology. Annu Rev Pathol.

[2] Senft D, Ronai ZA (2015). UPR, autophagy, and mitochondria crosstalk underlies the ER stress response. Trends Biochem Sci.

[3] Hetz C (2012). The unfolded protein response: controlling cell fate decisions under ER stress and beyond. Nat Rev Mol Cell Biol.

[4] Xu D, Liu L, Zhao Y, Yang L, Cheng J, Hua R (2020). Melatonin protects mouse testes from palmitic acid-induced lipotoxicity by attenuating oxidative stress and DNA damage in a SIRT1-dependent manner. J Pineal Res.

[5] Li C, Zhang K, Pan G, Ji H, Li C, Wang X (2021). Dehydrodiisoeugenol inhibits colorectal cancer growth by endoplasmic reticulum stress-induced autophagic pathways. J Exp Clin Cancer Res.

[6] Urra H, Dufey E, Avril T, Chevet E, Hetz C (2016). Endoplasmic reticulum stress and the hallmarks of cancer. Trends Cancer.

[7] Liu X, Chen Y, Wang H, Wei Y, Yuan Y, Zhou Q (2021). Microglia-derived IL-1β promoted neuronal apoptosis through ER stress-mediated signaling pathway PERK/eIF2α/ATF4/CHOP upon arsenic exposure. J Hazard Mater.

[8] Tao SC, Yuan T, Rui BY, Zhu ZZ, Guo SC, Zhang CQ (2017). Exosomes derived from human platelet-rich plasma prevent apoptosis induced by glucocorticoid-associated endoplasmic reticulum stress in rat osteonecrosis of the femoral head via the Akt/Bad/Bcl-2 signal pathway. Theranostics.

[9] Ren JL, Chen Y, Zhang LS, Zhang YR, Liu SM, Yu YR (2021). Intermedin(1-53) attenuates atherosclerotic plaque vulnerability by inhibiting CHOP-mediated apoptosis and inflammasome in macrophages. Cell Death Dis.

[10] Wang Z, Liu P, Inuzuka H, Wei W (2014). Roles of F-box proteins in cancer. Nat Rev Cancer.

[11] Peters JM (2003). Emi1 proteolysis: how SCF(beta-Trcp1) helps to activate the anaphase-promoting complex. Mol Cell.

[12] Cappell SD, Mark KG, Garbett D, Pack LR, Rape M, Meyer T (2018). EMI1 switches from being a substrate to an inhibitor of APC/C(CDH1) to start the cell cycle. Nature.

[13] Zhang Y, Jost M, Pak RA, Lu D, Li J, Lomenick B (2022). Adaptive exchange sustains cullin-RING ubiquitin ligase networks and proper licensing of DNA replication. Proc Natl Acad Sci USA.

[14] Yim H, Erikson RL (2009). Polo-like kinase 1 depletion induces DNA damage in early S prior to caspase activation. Mol Cell Biol.

[15] Ballabeni A, Park IH, Zhao R, Wang W, Lerou PH, Daley GQ (2011). Cell cycle adaptations of embryonic stem cells. Proc Natl Acad Sci USA.

[16] Roy Burman SS, Nance ML, Jeliazkov JR, Labonte JW, Lubin JH, Biswas N (2020). Novel sampling strategies and a coarse-grained score function for docking homomers, flexible heteromers, and oligosaccharides using Rosetta in CAPRI rounds 37-45. Proteins.

[17] Wang K, Qu X, Liu S, Yang X, Bie F, Wang Y (2018). Identification of aberrantly expressed F-box proteins in squamous-cell lung carcinoma. J Cancer Res Clin Oncol.

[18] Marze NA, Roy Burman SS, Sheffler W, Gray JJ (2018). Efficient flexible backbone protein-protein docking for challenging targets. Bioinformatics.

[19] Wu Y, Li X, Jia J, Zhang Y, Li J, Zhu Z (2018). Transmembrane E3 ligase RNF183 mediates ER stress-induced apoptosis by degrading Bcl-xL. Proc Natl Acad Sci USA.

[20] Zheng S, Wang X, Liu H, Zhao D, Lin Q, Jiang Q (2023). iASPP suppression mediates terminal UPR and improves BRAF-inhibitor sensitivity of colon cancers. Cell Death Differ.

[21] Clijsters L, Hoencamp C, Calis JJA, Marzio A, Handgraaf SM, Cuitino MC (2019). Cyclin F controls cell-cycle transcriptional outputs by directing the degradation of the three activator E2Fs. Mol Cell.

[22] Chen HZ, Tsai SY, Leone G (2009). Emerging roles of E2Fs in cancer: an exit from cell cycle control. Nat Rev Cancer.

[23] Attwooll C, Lazzerini Denchi E, Helin K (2004). The E2F family: specific functions and overlapping interests. Embo J..

[24] Jang SM, Redon CE, Thakur BL, Bahta MK, Aladjem MI (2020). Regulation of cell cycle drivers by Cullin-RING ubiquitin ligases. Exp Mol Med.

[25] Yan B, Li X, Peng M, Zuo Y, Wang Y, Liu P (2023). The YTHDC1/GLUT3/RNF183 axis forms a positive feedback loop that modulates glucose metabolism and bladder cancer progression. Exp Mol Med.

[26] Seo SU, Woo SM, Im SS, Jang Y, Han E, Kim SH (2022). Cathepsin D as a potential therapeutic target to enhance anticancer drug-induced apoptosis via RNF183-mediated destabilization of Bcl-xL in cancer cells. Cell Death Dis.

[27] Clifton LA, Wacklin-Knecht HP, Ådén J, Mushtaq AU, Sparrman T, Gröbner G (2023). Creation of distinctive Bax-lipid complexes at mitochondrial membrane surfaces drives pore formation to initiate apoptosis. Sci Adv.

