# Supplementary material for: FBXO5-mediated RNF183 degradation prevents endoplasmic reticulum stress-induced apoptosis and promotes colon cancer progression
Source: Cell Death Dis. 2024 Jan 11;15(1):33. doi: 10.1038/s41419-024-06421-2 (PMC10784456; doi:10.1038/s41419-024-06421-2)
Supplement: Supplementary file 1 — supplementary data [file 41419_2024_6421_MOESM1_ESM.pptx]

## Slide 1
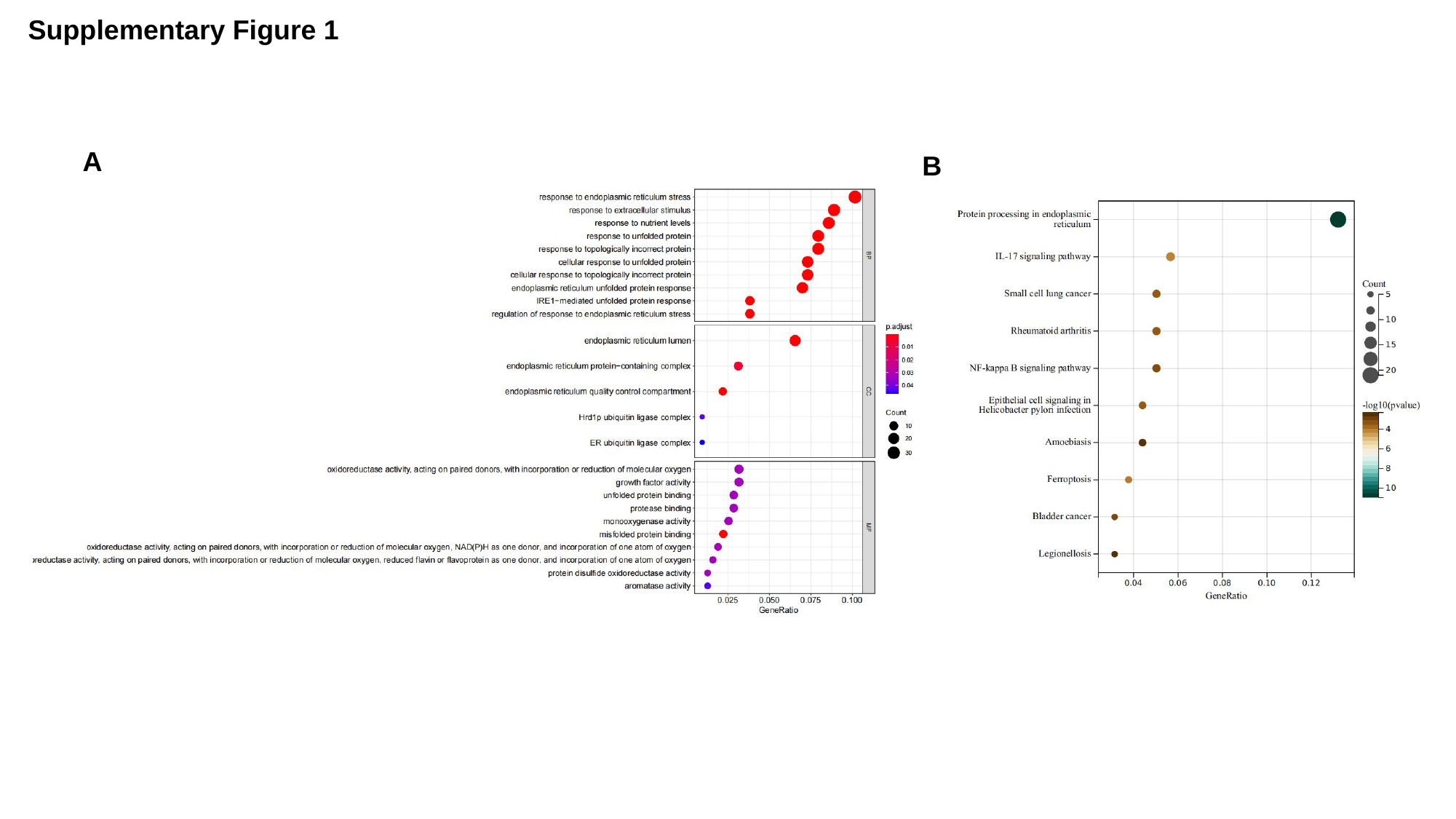

Supplementary Figure 1
A
B

## Slide 2
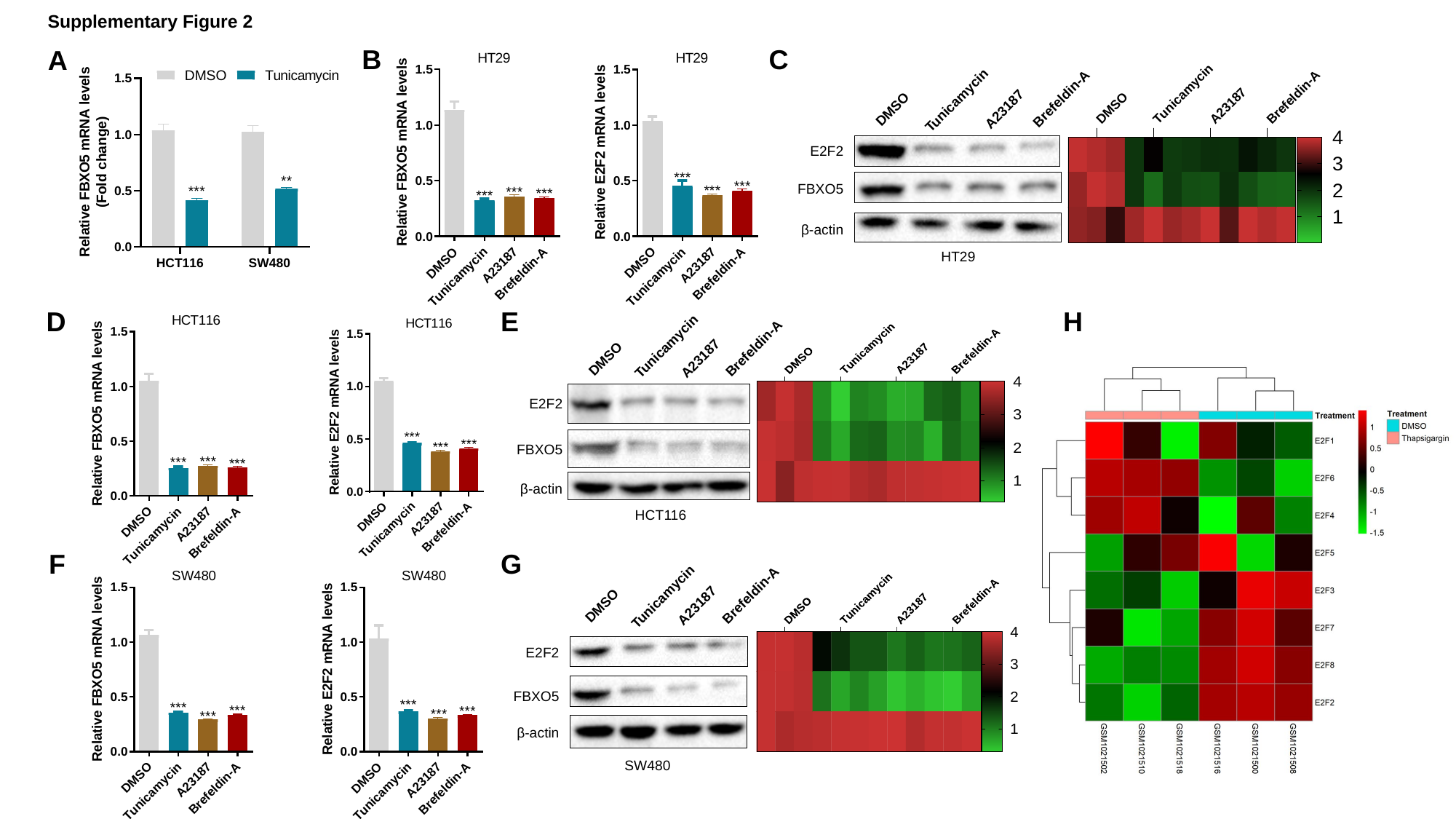

Supplementary Figure 2
B
C
A
Tunicamycin
A23187
Brefeldin-A
DMSO
E2F2
FBXO5
β-actin
HT29
H
D
E
Tunicamycin
A23187
Brefeldin-A
DMSO
E2F2
FBXO5
β-actin
HCT116
F
G
Tunicamycin
A23187
Brefeldin-A
DMSO
E2F2
FBXO5
β-actin
SW480

## Slide 3
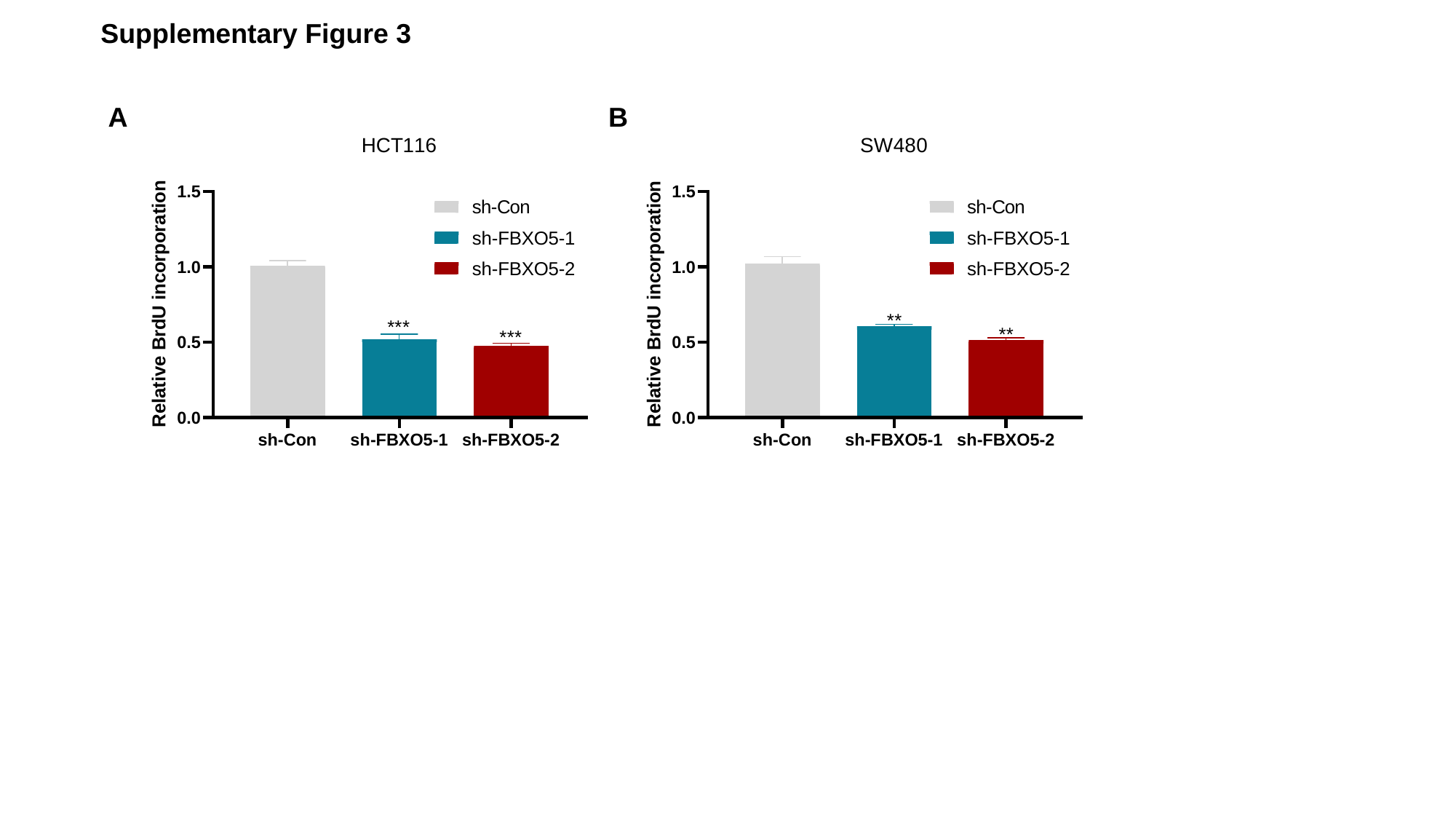

Supplementary Figure 3
A
B

## Slide 4
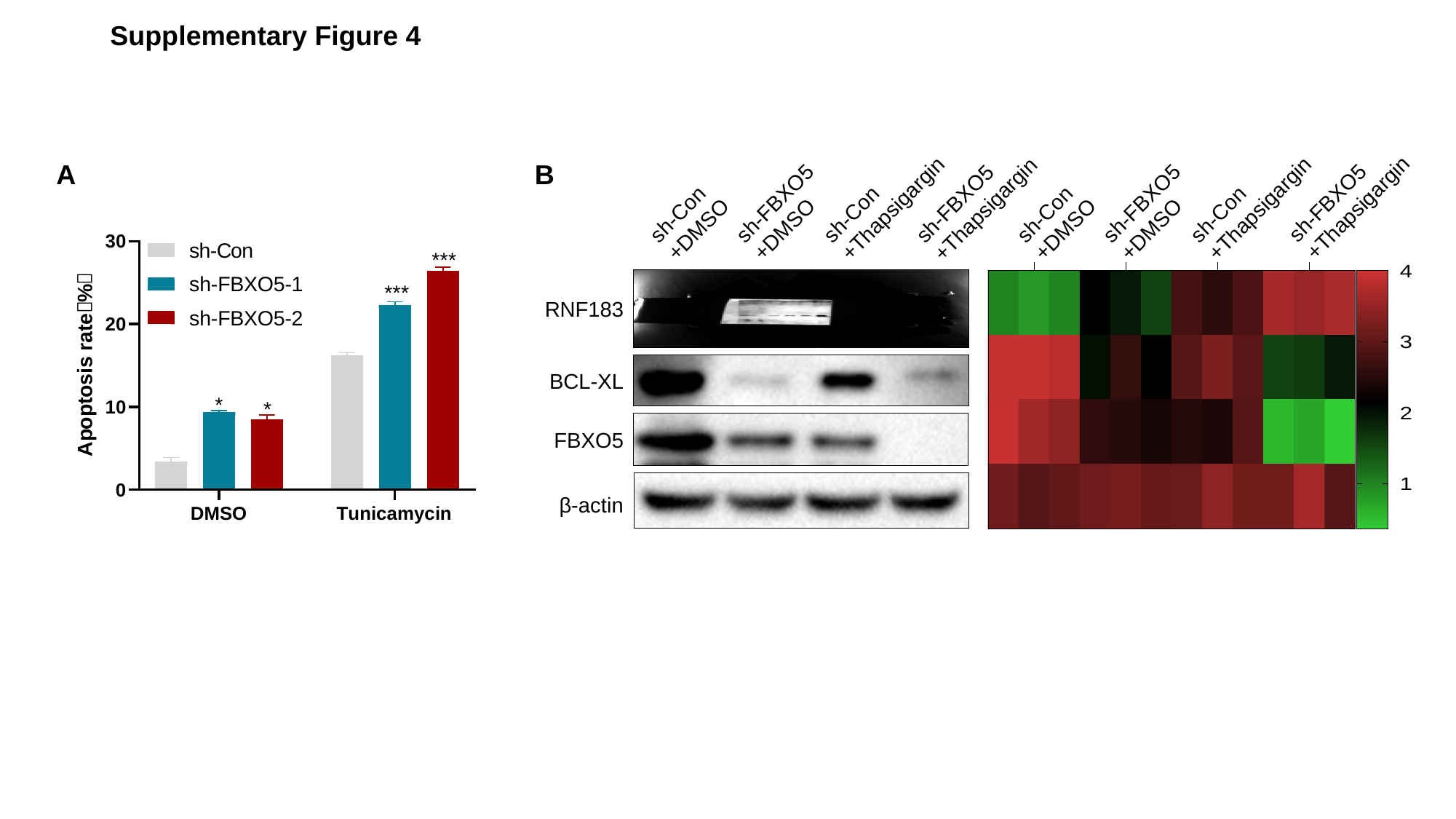

Supplementary Figure 4
A
B
sh-Con
+Thapsigargin
sh-Con
+Thapsigargin
sh-FBXO5
+Thapsigargin
sh-FBXO5
+Thapsigargin
sh-Con
+DMSO
sh-FBXO5
+DMSO
sh-Con
+DMSO
sh-FBXO5
+DMSO
RNF183
BCL-XL
FBXO5
β-actin
