# Supplementary material for: FBXO5-mediated RNF183 degradation prevents endoplasmic reticulum stress-induced apoptosis and promotes colon cancer progression
Source: Cell Death Dis. 2024 Jan 11;15(1):33. doi: 10.1038/s41419-024-06421-2 (PMC10784456; doi:10.1038/s41419-024-06421-2)
Supplement: Supplementary file 8 — Supplementary Figure legend [file 41419_2024_6421_MOESM8_ESM.docx]

**Supplementary data**

**Supplementary Figure 1** The GO and KEGG analysis of the significantly up-regulated genes in response to ER-stress

1. GO analysis of the significantly upregulated genes for biological process (BP), cellular compartment (CC), and molecular function (MF).
2. KEGG pathway analysis of the significantly upregulated genes.

**Supplementary Figure 2** ER-stress reduced the expression of FBXO5 and E2F family members

1. Relative mRNA fold change of FBXO5 in HCT116 cells or SW480 cells treated with 1μM tunicamycin for 12 hr. *^**^p<0.01 vs* DMSO*, ^***^p<0.001 vs* DMSO*.*
2. Relative mRNA fold change of FBXO5 and E2F2 in HT29 cells treated with ER stress inducers (tunicamycin(1μM), A23187(1μM) and Brefeldin-A(10μM)) for 12 hr. *^***^p<0.001 vs* DMSO*.*
3. Immunoblot analysis of FBXO5 and E2F2 in HT29 cells treated with 1μM ER stress inducers (tunicamycin(1μM), A23187(1μM) and Brefeldin-A(10μM)) for 12 hr. The quantitative information of each protein of the triplicate experiments was obtained by Image J software and displayed by the heatmap on the right side.
4. Relative mRNA fold change of FBXO5 and E2F2 in HCT116 cells treated with ER stress inducers in B for 12 hr. *^***^p<0.001 vs* DMSO*.*
5. Immunoblot analysis of FBXO5 and E2F2 in HCT116 cells treated with 1μM ER stress inducers in B for 12 hr. The quantitative information of each protein of the triplicate experiments was obtained by Image J software and displayed by the heatmap on the right side.
6. Relative mRNA fold change of FBXO5 and E2F2 in SW480 cells treated with 1μM ER stress inducers in B for 12 hr. *^***^p<0.001 vs* DMSO*.*
7. Immunoblot analysis of FBXO5 and E2F2 in SW480 cells treated with 1μM ER stress inducers in B for 12 hr. The quantitative information of each protein of the triplicate experiments was obtained by Image J software and displayed by the heatmap on the right side.
8. Heatmap of clustering analysis showed the expression of E2F members (with 8 quantifiable members) following thapsigargin treatment.

**Supplementary Figure 3** The mRNA levels of FBXO5 in colon cancer cells with FBXO5 silencing

1. Relative BrdU incorporation of FBXO5 in HCT116 cells after stable knockdown of FBXO5 by two different shRNAs. *^***^p<0.001 vs* sh-Con*.*
2. Relative BrdU incorporation of FBXO5 in SW480 cells after stable knockdown of FBXO5 by two different shRNAs. *^**^p<0.01 vs* sh-Con*.*

**Supplementary Figure 4** FBXO5 prevents ER stress-induced apoptosis of colon cancer cells

1. Quantification of apoptotic HCT116 cells stable knockdown of FBXO5 with or without 1μM tunicamycin treatment for 12 hr and analyzed by flow cytometry. *^*^p<0.05 vs* sh-Con*, ^***^p<0.001 vs* sh-Con*.*
2. Transfection of control sh-RNA or E2F2 sh-RNA in HCT116 cells treated with DMSO or thapsigargin, followed by immunoblotting with the indicated antibodies. The quantitative information of each protein of the triplicate experiments was obtained by Image J software and displayed by the heatmap on the right side.

**Supplementary Table 1** The list of thapsigargin-upregulated genes

**Supplementary Table 2** The list of thapsigargin-downregulated genes

**Supplementary Table 3** The list of F-box family member differential genes after thapsigargin treatment

**Supplementary Table 4** The co-expression gene list of FBXO5 in TCGA colon cancer tissues

**Supplementary Table 5** The gene list of potential FBXO5 upstream regulator

**Supplementary Table 6** The list of F-box family member differential genes in GSE62321 dataset
